# Supplementary material for: Exceptional energy harvesting from coupled bound states
Source: Nat Commun. 2025 Apr 13;16:3515. doi: 10.1038/s41467-025-58831-1 (PMC11994771; doi:10.1038/s41467-025-58831-1)
Supplement: Supplementary file 1 — Supplementary Information [file 41467_2025_58831_MOESM1_ESM.pdf]

Exceptional energy harvesting from coupled bound states
— Supplementary Information —

Felix Kronowetter*[1,2,3], Anton Melnikov[1], Marcus Maeder[1], Tao Yang[1], Yan Kei Chiang[2], Sebastian Oberst[3], David A. Powell[2], and Steffen Marburg[1]

[1]*Chair of Vibro-Acoustics of Vehicles and Machines, Department of Engineering Physics and Computation, Technical University of Munich, TUM School of Engineering and Design*

[2]*School of Engineering and Information Technology, University of New South Wales, Northcott Drive, Canberra, ACT 2600, Australia*

[3]*School of Mechanical and Mechatronic Engineering, Centre for Audio, Acoustics and Vibration, Faculty of Engineering and IT, University of Technology Sydney, Sydney, Australia*

S 1 Coupled mode theory

We use the coupled mode theory [1–3] to predict the location of the bound state in the continuum (BIC). We consider a reduced two-dimensional coupled rectangular waveguide-resonator system for simplicity (see Fig. S 1).

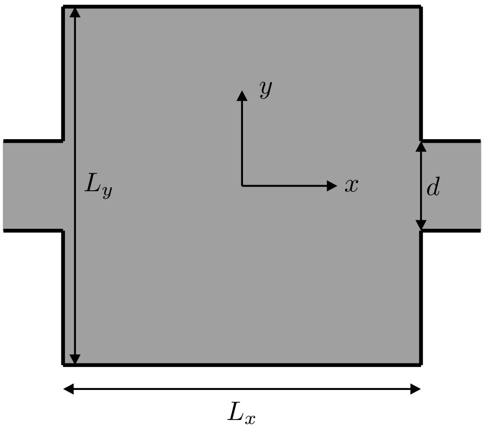

Fig. S 1. Schematic drawing of a coupled two-dimensional waveguide-resonator system.

We place the resonator's center at the origin, and the left and right waveguides connect along the x-axis. All formulas below assume that the origin of the coordinate system is in the center of the resonator. Here, the waveguide spans from $y = -1/2$ to $y = +1/2$. The first step is to compute the eigenfrequencies and eigenmodes of a closed resonator. The eigenfrequencies can be solved analytically with Neumann boundary conditions as follows [1, 2, 4]

$$E_{m,n} = \left(\frac{(m-1)}{L_x}\right)^2 + \left(\frac{(n-1)}{L_y}\right)^2, \quad \text{with} \quad m, n = 1, 2, 3, \ldots, \tag{1}$$

where $E_{m,n}$ is the "energy" of the system defined by the squared resonant angular frequency $\omega_0 = 2\pi f$ normalized by c, where c is the speed of sound in air and f is the resonant frequency. We obtain the corresponding modes, ψ, by

$$\psi_{m,n} = \sqrt{\frac{(2-\delta_m^1)(2-\delta_n^1)}{L_x L_y}} \cos\left(\frac{\pi(m-1)(2x-L_x)}{2L_x}\right) \cos\left(\frac{\pi(n-1)(2y-L_y)}{2L_y}\right), \tag{2}$$

*Corresponding Author: felix.kronowetter@tum.de

with δ_m^1 and δ_n^1 being the Kronecker delta. Furthermore, the dispersion relation of the waveguides follows as [5]:

$$E_p = k_p^2 + \frac{(p-1)^2\pi^2}{d^2}, \quad \text{with} \quad p = 1, 2, 3, \ldots, \tag{3}$$

where k_p denotes the wavenumber of the p-th channel of the waveguide. Furthermore, the propagating solutions in form of plane waves can be written as

$$\psi_{p,C} = \frac{1}{4\pi k_p} e^{\pm \mathrm{i} k_p x \pm \mathrm{i} k_p (\pm \frac{L_x}{2})} \phi_p, \quad \text{with} \quad C = L, R. \tag{4}$$

Here, the parameter, $C = L, R$, represents the left or right waveguide, respectively. The transverse modes ϕ of the waveguides are a solution to a waveguide with Neumann boundary conditions as

$$\phi_p = \frac{\sqrt{(2-\delta_p^1)}}{d} \cos\left(\frac{\pi(p-1)(2y+d)}{2d}\right), \tag{5}$$

with the pre-factor $\frac{\sqrt{(2-\delta_p^1)}}{d}$ ensuring the orthogonality of the transverse modes [6]. According to Maksimov et al. [1] and Pichugin et al. [7], the vectors $\boldsymbol{W}_{Cp}$ containing the coupling constants $W_{L;m,n;p}$ between eigenmodes of the closed resonator and p-th propagation channels of the left/right waveguide are evaluated as overlap integrals between the transverse waveguide modes and the cavity modes at the waveguide-cavity interface

$$W_{L;m,n;p} = \int_{-\frac{d}{2}}^{\frac{d}{2}} \psi_{m,n}(x = -\frac{L_x}{2}, y)\phi_p(x = -\frac{L_x}{2}, y)\,\mathrm{d}y, \tag{6}$$

$$W_{R;m,n;p} = \int_{-\frac{d}{2}}^{\frac{d}{2}} \psi_{m,n}(x = \frac{L_x}{2}, y)\phi_p(x = \frac{L_x}{2}, y)\,\mathrm{d}y. \tag{7}$$

The effective non-Hermitian Hamiltonian follows as [7–11]

$$\boldsymbol{H}_{eff} = \boldsymbol{H}_R - \sum_{p=1}^{\infty} \sum_{C=L,R} \mathrm{i} k_p \boldsymbol{W}_{C,p} \boldsymbol{W}_{C,p}^{\dagger}, \tag{8}$$

where the † symbol stands for the Hermitian transpose. The BIC is perfectly decoupled from the continuum. Therefore, its eigenfunction must be given by

$$\int_{-\frac{d}{2}}^{\frac{d}{2}} \psi_{BIC}(x = \pm\frac{L_x}{2}, y))\,\mathrm{d}y = 0. \tag{9}$$

In principle, it is possible to decompose the eigenfunction of any BIC as

$$\psi_{BIC} = \sum_{m,n} \chi_{m,n} \psi_{m,n}(x, y). \tag{10}$$

Finaly, we find the unknowns, $\chi_{m,n}$, by solving the eigenvalue problem based on the scattering function of the system [1, 2]

$$(\boldsymbol{H}_{eff} - \lambda^2 \boldsymbol{I})\boldsymbol{\chi} = 0. \tag{11}$$

Only the first propagation channel of the waveguide $p = 1$ is of interest. As can be seen from Eq. (8) and Eq. (11), the eigenvalue problem is not linear since the $\boldsymbol{H}_{eff}$ depends on the wavenumber of the waveguides, which links the eigenvalue via the dispersion relation. Furthermore, we solve the eigenvalue problem by determining the characteristic polynomial and identifying its roots. Since two strongly coupled degenerate modes form a Friedrich-Wintgen BIC, it is reasonable to use a two-mode approximation of the system. The two-level effective non-Hermitian Hamiltonian reads as

$$\boldsymbol{H}_{eff} = \begin{bmatrix} E_{m,n} & 0 \\ 0 & E_{n,m} \end{bmatrix} - \mathrm{i}2k_1 \begin{bmatrix} W_{m,n}^2 & W_{m,n}W_{n,m} \\ W_{n,m}W_{m,n} & W_{n,m}^2 \end{bmatrix}. \tag{12}$$

The factor 2 in front of the coupling matrix is because the coupling constants for the left and right waveguides are identical $W_{L;m,n} = W_{R;m,n}$ in the formulation used here. The two-level effective non-Hermitian Hamiltonian has a real eigenvalue associated with the BIC. The corresponding eigenvector reads as [2]

$$\boldsymbol{\chi} = \frac{W_{m,n}W_{n,m}}{\sqrt{W_{m,n}^2 + W_{n,m}^2}} \left(\frac{1}{W_{m,n}}, \frac{-1}{W_{n,m}} \right)^{\dagger}. \tag{13}$$

With reference to Eq. (10), we approximate the BIC mode as

$$\psi_{BIC} \approx \frac{W_{n,m}}{\sqrt{W_{m,n}^2 + W_{n,m}^2}} \psi_{m,n} - \frac{W_{m,n}}{\sqrt{W_{m,n}^2 + W_{n,m}^2}} \psi_{n,m}. \tag{14}$$

All the above equations can be adapted to apply mirror symmetry. Thus, only the left waveguide is considered and the integratable domain is reduced. Figure S 2 shows the first reduction step.

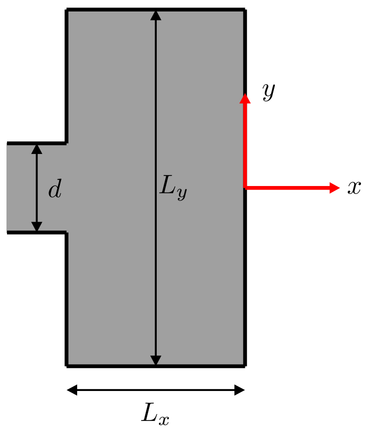

Fig. S 2. Schematic drawing of a coupled two-dimensional waveguide-resonator system with mirror symmetry.

All of the subsequent reduction steps work in a similar way.

S 2 Numerical prediction of FRC cavity

Figure. S 3 shows maximum pressure and voltage output data over a frequency range from $f = 2\,\text{kHz}$ to $f = 2.4\,\text{kHz}$ and varying cavity lengths from $L_x = 65\,\text{mm}$ to $L_x = 85\,\text{mm}$.

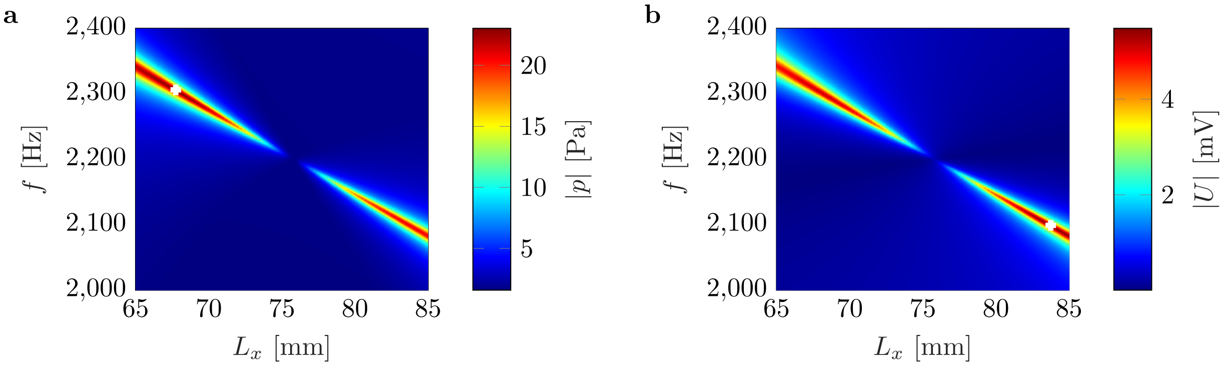

Fig. S 3. FRC Mappings. a Determination of the maximum absolute pressure inside the cavity (indicated by the white plus sign) using parameter sweeps over cavity length and frequency. **b** Determination of the maximum voltage output (indicated by the white plus sign) using parameter sweeps over cavity length and frequency.

The maximum voltage output is found at a cavity length of $L_x = 83.5\,\text{mm}$.

S 3 Experimental data

Pictures of the sound hard reference and the Helmholtz resonator, both with mounted PZT, visualizes Fig. S 4.

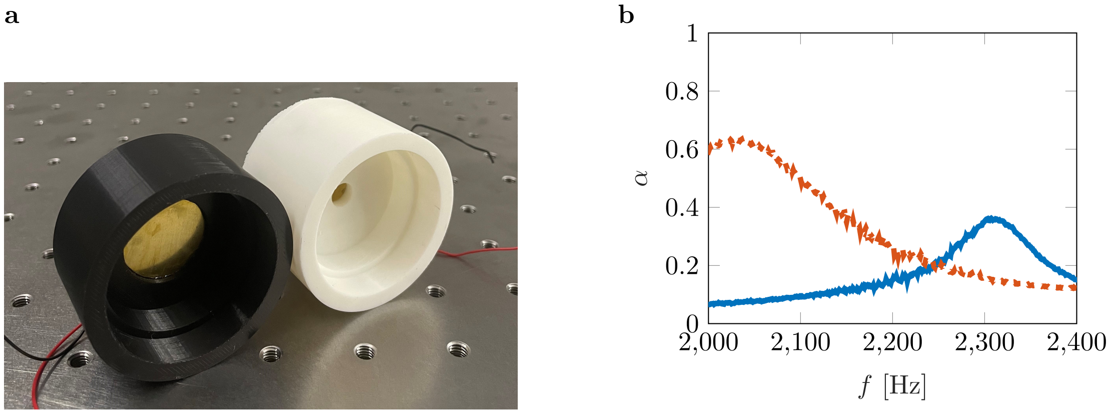

Fig. S 4. Impedance tube. a Printed samples of the sound hard reference and the Helmholtz resonator with mounted PZTs. **b** Results of the impedance tube measurements for the PZT (solid blue line) and the Helmholtz resonator without PZT (dashed orange line)..

Figure S 5 provides the velocity mappings of the PZT excited with a pseudo white noise signal and a sinusoidal excitation corresponding to the peak velocity from the spectral excitation. More concretely, Figs. S 5a and 5b both show concentric velocity distributions, indicating that the PZTs are correctly mounted on the sample.

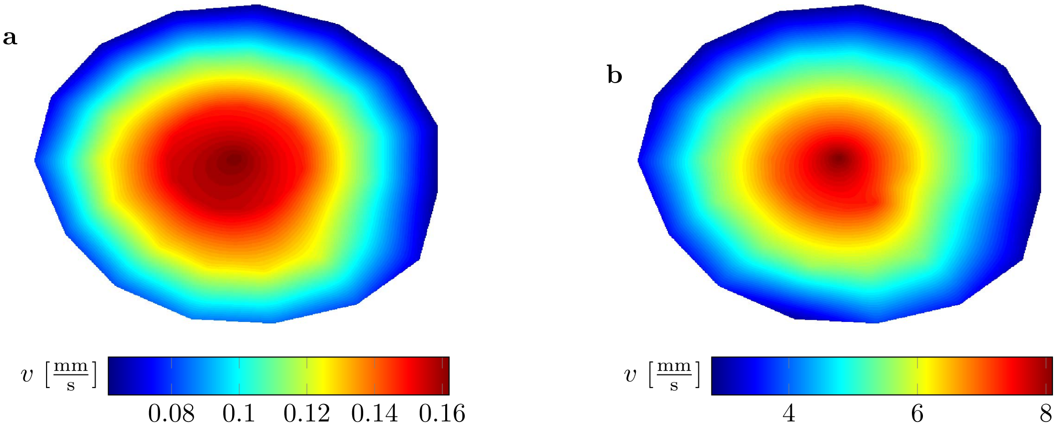

Fig. S 5. LDV velocity data. a Visualization of the velocity of the PZT by means of an LDV under white noise excitation. **b** Visualization of the velocity of the PZT using an LDV excited at the target frequency of $f = 2068.5\,\mathrm{Hz}$.

S 4 Coupled-BICs

We carry out a parameter study to determine the configuration of the two coupled BICs with maximum pressure enhancement. The frequency, cavity length, and distance between the FRCs are varied. They range from $f = 1900\,\text{Hz}$ to $f = 2400\,\text{Hz}$ in $2\,\text{Hz}$ steps, $L_{x,2} = 64\,\text{mm}$ to $L_{x,2} = 90\,\text{mm}$ in $2\,\text{mm}$ steps, and $\Delta = 40\,\text{mm}$ to $\Delta = 100\,\text{mm}$ in $2\,\text{mm}$ steps, respectively. A plane wave with a pressure of $1\,\text{Pa}$ excites the system, with the corresponding results in Fig. S 6 for $\Delta = 72\,\text{mm}$. The black cross indicates the Fano peak of the configuration

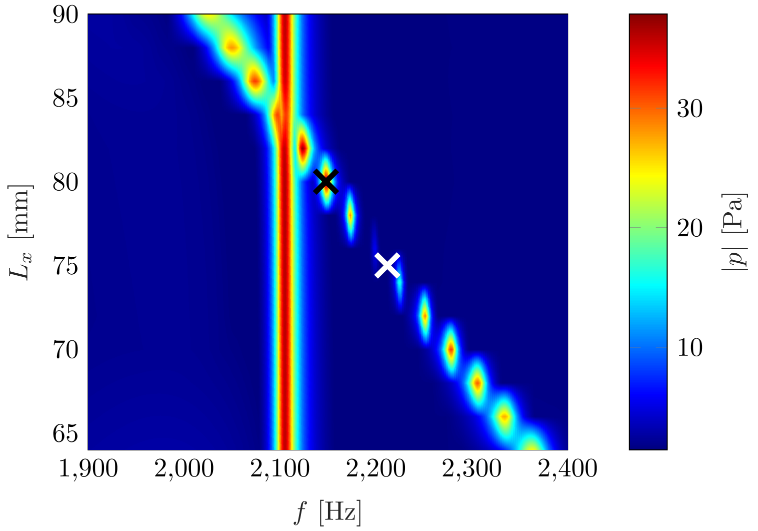

Fig. S 6. Coupled BICs. Mapping of the maximum pressure enhancement of the coupled FRCs in terms of frequency, cavity length, and distance ranging from $f = 1900\,\text{Hz}$ to $f = 2400\,\text{Hz}$ in $2\,\text{Hz}$ steps, $L_{x,2} = 64\,\text{mm}$ to $L_{x,2} = 90\,\text{mm}$ in $2\,\text{mm}$ steps, and $\Delta = 72\,\text{mm}$, respectively.

with the highest pressure enhancement. A pressure maximum of $38\,\text{Pa}$ occurs at $L_{x,2} = 80\,\text{mm}$ and $\Delta = 72\,\text{mm}$. The white cross indicates the disappearing Fano peak and, therefore, an additional BIC.

S 5 Optimum resistance study

We carry out an optimum resistance study to determine which resistance will give the highest output power P_o from the FRC harvester. The output power for different resistances from $10\,\Omega$ to $10\,\mathrm{k}\Omega$ is shown in Fig. S 7. The maximum output power is achieved around $2511\,\Omega$. The numerical results are an approximation of the

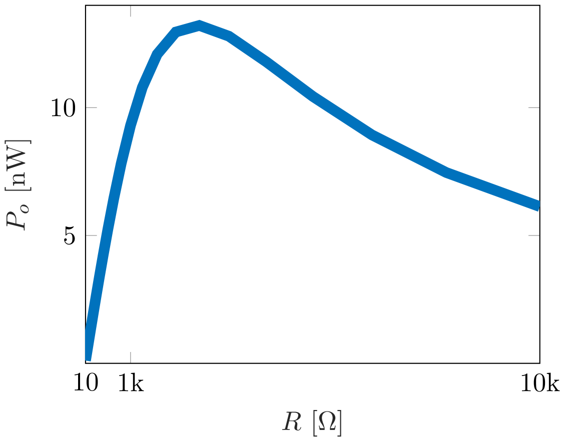

Fig. S 7. Optimum resistance analysis. FRC harvester output power over a resistance range from $10\,\Omega$ to $10\,\mathrm{k}\Omega$.

PZT used in the experimental setup, as we can only estimate its material parameters. Thus, the order of $1\,\mathrm{k}\Omega$ is expected for the maximum output power of the experimental setup.

References

[1] D. Maksimov, A. Sadreev, A. A. Lyapina, and A. Pilipchuk. "Coupled mode theory for acoustic resonators". In: *Wave Motion* 56 (Feb. 2015). DOI: 10.1016/j.wavemoti.2015.02.003.

[2] A. A. Lyapina, D. Maksimov, A. Pilipchuk, and A. Sadreev. "Bound states in the continuum in open acoustic resonators". In: *Journal of Fluid Mechanics* 780 (June 2015). DOI: 10.1017/jfm.2015.480.

[3] L. Huang et al. "General Framework of Bound States in the Continuum in an Open Acoustic Resonator". In: *Phys. Rev. Applied* 18 (5 Nov. 2022), p. 054021. DOI: 10.1103/PhysRevApplied.18.054021.

[4] A. Rona. "The Acoustic Resonance of Rectangular and Cylindrical Cavities". In: *Journal of Algorithms & Computational Technology* 1 (May 2007). DOI: 10.1260/174830107782424110.

[5] M. Möser. "Schallabsorption". In: *Technische Akustik*. Berlin, Heidelberg: Springer Berlin Heidelberg, 2015, pp. 183–232. DOI: 10.1007/978-3-662-47704-5_6.

[6] V. Pagneux. "Trapped Modes and Edge Resonances in Acoustics and Elasticity". In: *Dynamic Localization Phenomena in Elasticity, Acoustics and Electromagnetism*. Ed. by R. V. Craster and J. Kaplunov. Vienna: Springer Vienna, 2013, pp. 181–223. DOI: 10.1007/978-3-7091-1619-7_5.

[7] K. Pichugin, H. Schanz, and P. Šeba. "Effective coupling for open billiards". In: *Phys. Rev. E* 64 (5 Oct. 2001), p. 056227. DOI: 10.1103/PhysRevE.64.056227.

[8] F.-M. Dittes. "The decay of quantum systems with a small number of open channels". In: *Physics Reports* 339 (Dec. 2000), pp. 215–316. DOI: 10.1016/S0370-1573(00)00065-X.

[9] J. Okolowicz, M. Płoszajczak, and I. Rotter. "Dynamics of quantum systems embedded in a continuum". In: *Physics Reports* 374 (Feb. 2003), pp. 271–383. DOI: 10.1016/S0370-1573(02)00366-6.

[10] A. F. Sadreev and I. Rotter. "S-matrix theory for transmission through billiards in tight-binding approach". In: *Journal of Physics A: Mathematical and General* 36.45 (Oct. 2003), p. 11413. DOI: 10.1088/0305-4470/36/45/005.

[11] A. F. Sadreev. "Interference traps waves in open system: Bound states in the continuum". In: *Reports on Progress in Physics* 84 (Mar. 2021). DOI: 10.1088/1361-6633/abefb9.
